# Understanding spearfishing in a coral reef fishery: Fishers’ opportunities, constraints, and decision-making

**DOI:** 10.1371/journal.pone.0181617

**Published:** 2017-07-27

**Authors:** Tyler Pavlowich, Anne R. Kapuscinski

**Affiliations:** Environmental Studies Program, Dartmouth College, Hanover, New Hampshire, United States of America; Department of Agriculture and Water Resources, AUSTRALIA

## Abstract

Social and ecological systems come together during the act of fishing. However, we often lack a deep understanding of the fishing process, despite its importance for understanding and managing fisheries. A quantitative, mechanistic understanding of the opportunities fishers encounter, the constraints they face, and how they make decisions within the context of opportunities and constraints will enhance the design of fisheries management strategies to meet linked ecological and social objectives and will improve scientific capacity to predict impacts of different strategies. We examined the case of spearfishing in a Caribbean coral reef fishery. We mounted cameras on fishers’ spearguns to observe the fish they encountered, what limited their ability to catch fish, and how they made decisions about which fish to target. We observed spearfishers who dove with and without the assistance of compressed air, and compared the fishing process of each method using content analysis of videos and decision models of fishers’ targeting selections. Compressor divers encountered more fish, took less time to catch each fish, and had a higher rate of successful pursuits. We also analyzed differences among taxa in this multispecies fishery, because some taxa are known to be ecologically or economically more valuable than others. Parrotfish are ecologically indispensable for healthy coral reefs, and they were encountered and captured more frequently than any other taxon. Fishers made decisions about which fish to target based on a fish’s market value, proximity to the fisher, and taxon. The information uncovered on fishers’ opportunities, constraints, and decision making has implications for managing this fishery and others. Moreover, it demonstrates the value of pursuing an improved understanding of the fishing process from the perspective of the fishers.

## Introduction

Fishing is an act in which society and ecosystems mix. Fishery management nearly always intervenes in the act of fishing in order to achieve ecological outcomes (e.g., maintaining the abundances of species with different functional roles in the ecosystem), social outcomes (e.g., providing livelihood stability for fishers), or a mix of ecological and social outcomes. However, fisheries scientists and managers who aim to inform and implement management often lack a deep understanding of the process of fishing [1, 2]. The process of fishing is characterized not only by gear type, number of boats and catch statistics, but also by the opportunities, constraints and decisions that fishers face. For instance, the commonly used metric of catch per unit effort is the aggregate outcome of many interactions between a fisher and the fishery ecosystem [3]. These interactions and human decisions driving these interactions remain a black box for many fisheries. Researchers should seek a mechanistic understanding of the fishing process, specifically the way fishers experience the resource, the factors that control their ability to catch fish, and how they make decisions while fishing. Decisions made at the level of the individual fisher aggregate to determine fleet dynamics, which in turn has important consequences for ecological and social outcomes [1, 4].

An intimate understanding of fishing is needed for scientists and fisheries managers to develop effective management strategies, especially for fisheries where fishers have substantial flexibility in how they fish [5]. Fishers in small-scale fisheries often use multiple gears and catch multiple species [6, 7], a strategy that provides increased stability for their catch in the face of variability and uncertainty. This flexibility is advantageous for fishers, but it makes predicting the benefits and costs of alternative management interventions difficult [8]. Moreover, we cannot know how competing management interventions will affect the catch if we do not know how fishers will react to gear, species, or size restrictions. These knowledge gaps undermine capacity to quantitatively assess if an intervention will meet ecological objectives and how it will impact fishers [2]. Low capacity to predict outcomes of a management intervention poses the risk of unreasonable expectations, unexpected hardship, and resistance to or failure of the management process, especially in small-scale, data-poor fisheries.

**Fig 1:**
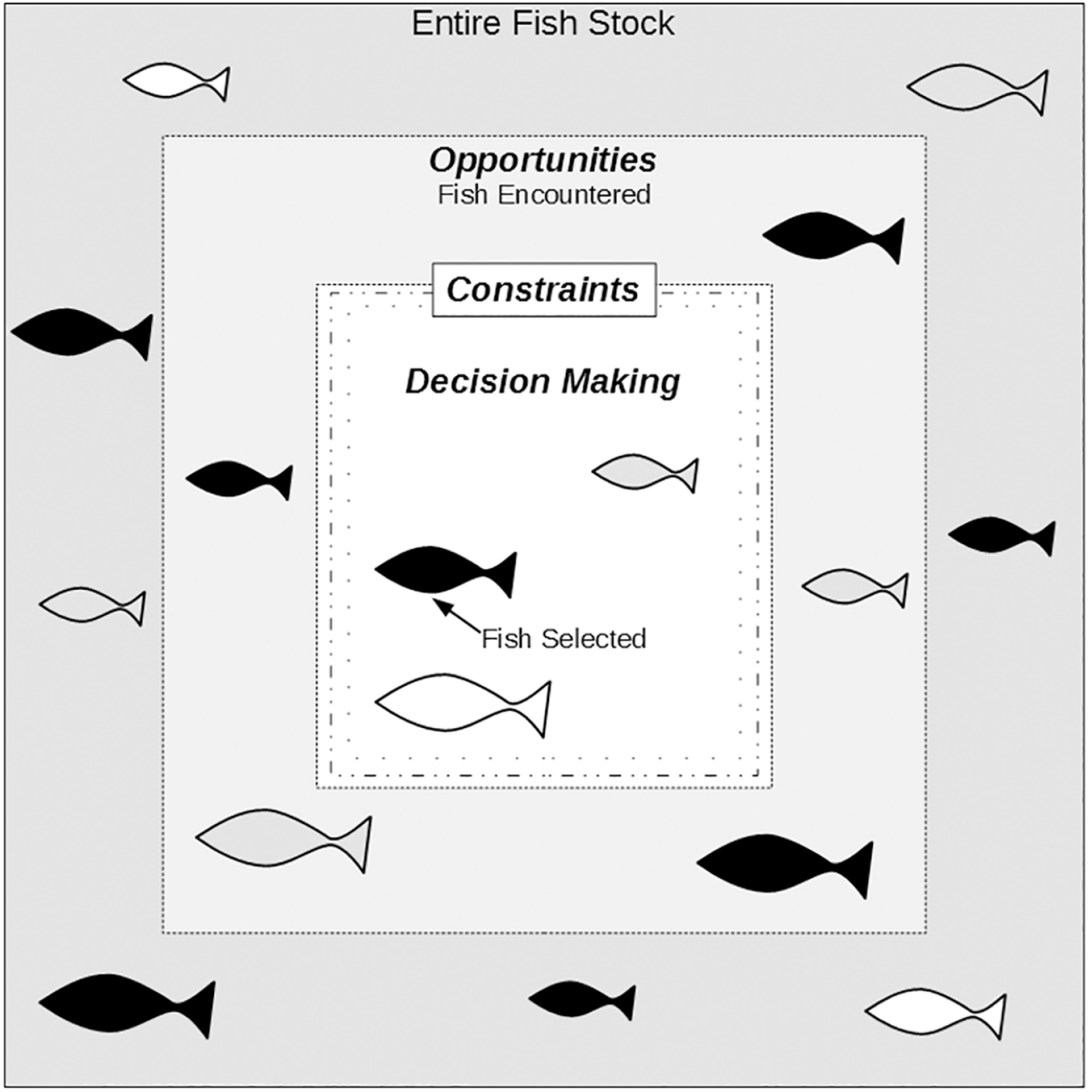
Heuristic of the context of spearfishing. The fish available to fishers are a subset of the entire stock, limited by which fish they actually encounter, and further limited by constraints on the fishers’ ability to catch them.

Fishers make decisions about how to fish within a context of opportunities and constraints, as well as in response to incentives from the market or their subsistence needs[4]. In this study, we consider opportunities as the fish each fisher encounters which define the possibilities of what they can catch. Constraints are any factors that shrink the possible catch. These factors include how long it takes to catch fish, the feasible length of a fishing trip, the number of fish that a fisher can capture at one time, and the ability to successfully capture a pursued fish. Presented with both opportunities and constraints, fishers decide which fish to target, and this decision making further influences catch characteristics. Together, opportunities, constraints, and targeting decisions all affect the quantity and type of fish in fishers’ catch (Fig 1).

Spearfishing is a common form of fishing in artisanal fisheries [9–12], that has been shown to strongly impact fish communities [13–16]. Perhaps no other fishing method allows fishers as much selectivity as spearfishing. Spearfishers individually select every fish they want to capture minute by minute or even second by second. Therefore, researchers must utilize underwater observations of the fishing process to collect data on the fish encountered, the constraints on fishers, how fish respond to fishers, and how fishers’ selection decisions influenced what they ultimately caught. Prior studies have examined fisher decision making with respect to gear choice, fishing location, and target species. These studies involved gear types and situations in which fishers make decisions about type of fish to pursue or how much effort to expend on time scales of tens of minutes [17], days [18–20], and days per month [21]. In our study, we mounted high definition underwater cameras to fishers’ spearguns in order to observe the fishing process from the fisher’s perspective and at a fine timescale of second by second to minute by minute. Bulleri and Benedetti-Cecchi [22] used a similar technique to conduct fish community surveys by watching videos of recreational fishing by spearfishers. Our study used this technique to observe the fish community and artisanal spearfishers’ actions. This is the first, to our knowledge, to examine the context and process of spearfishing, *per se*, and spearfishers’ behavior at the timescale that matches the scale at which fishers’ make decisions.

The research presented here focused on spearfishing in a coral reef fishery. Coral reefs and their accompanying fishery resources have been degraded globally, in part due to heavy exploitation by artisanal fishers [23–26]. Reefs around the world show patterns of depletion in the abundance and biomass of species with important ecological roles, namely herbivores and top predators [27–29]. Therefore, management goals often include increasing the abundance and biomass of critical functional groups [30–32]. Marine reserves and catch limits of certain species or sizes are common examples of management interventions aimed at improving ecosystem health and fishery productivity[33–35]. These kinds of interventions limit fishers’ ability to catch fish, which can have direct impacts on their wellbeing in communities with a strong dependence on fishery resources for people’s livelihoods [36, 37]. High dependence can also make compliance with restrictions more challenging. Therefore, there exists a moral obligation and a strategic motivation to have a good understanding of possible effects of any management action before implementation, to inform stakeholders of plausible positive and negative outcomes, and to navigate management options thoughtfully. Here, we seek to advance mechanistic understanding of fishers’ practices and decisions in the spearfishing process and to assess implications of this understanding for the management of spearfishing fisheries.

We pursued three objectives in order to better understand the fishing process:1) Quantify and describe the opportunities fishers encounter; 2) Identify constraints to the number and types of fish that can be caught while spearfishing; 3) Determine which fish characteristics influence fishers’ targeting decisions and the direction and magnitude of their effect.

Our results provide a novel look at the process of spearfishing in an artisanal coral reef fishery. Within each aspect of the fishing process, we base our analyses and interpretation of the results on two additional topics that correspond to pertinent management issues in coral reef fisheries: compressor (‘hookah’) diving versus freediving, and controlling the taxonomic composition of fishers’ catch. The approach we present to analyzing opportunities, constraints and decision making in the fishing process is adaptable to other fishing systems. Lastly, we discuss below how improved understanding of these fishing-process components can inform sustainable fishery management.

## Methods

### Study site

The rural village of Buen Hombre is located within Montecristi National Park in the northwest Dominican Republic. Men in the community fish the coastal coral reefs and seagrass beds as their main livelihood activity. Fishers mostly use rifle style spearguns, though some people use handlines, gillnets, and fish traps. There were approximately 40 divers regularly fishing in the community at the time of this study. People who freedive use only a mask, fins, a snorkel and speargun, and typically fish in waters less than 10 meters deep. Compressor divers use a mask, fins, speargun, and a regulator supplied with compressed air from a boat at the surface. This allows compressor divers to fish in deeper waters, typically between 10 and 35 m. Both freedivers and compressor divers use the same type of spearguns. From our previous work in this fishery, we knew that compressor divers capture more per person than freedivers, and that parrotfish comprise over half of the total catch (see Supplemental Information). Today, it is one of several villages in Montecristi Province that are part of a collaborative effort to improve marine resource management between the Dominican government, local nongovernmental organizations, and international environmental conservation and sustainable development organizations [12]. In collaboration with a Dominican nongovernmental organization, AgroFrontera, we began researching the social-ecological dynamics of the fishing system in Buen Hombre during the summer of 2012 [36,38]. Permission to conduct non-destructive, observational field studies on coastal marine ecosystems was granted to AgroFrontera by the Ministry of the Environment and Natural Resources of the Dominican Republic (permission #000200). In addition to ecological and fishing data, we performed participant observation and formal and informal interviews with fishers and other members of the community that provided the motivation and contextual and anecdotal information for this study. We obtained consent from the community and individual participants in a two-step process. First, we presented our general research agenda to a fishers’ meeting upon arriving in the community, and explicitly communicated that their participation was welcome but entirely voluntary and anonymous. Second, prior to each specific data-collection observation, we asked fishers if they wanted to participate, and told them that they were not obligated to and could withdraw their participation at any time. Consent was obtained verbally so as not to offend individuals who may have been uncomfortable reading and writing. Consent to participate was documented on the data collection sheets. Permission to conduct observational studies with human participants was not required from the Dominican government. This protocol, including the procedure to attain consent, was reviewed and accepted by the Committee for the Protection of Human Subjects at Dartmouth College (permission #24413).

### Data collection

The data for this study were collected in June and July of 2013 and 2014. Direct observations of spearfishers’ fishing activity were collected by attaching a compact video camera (GoPro Hero 3 Black) to the front end of the stock of fishers’ spearguns, facing forward. Fishers started recording videos when they entered the water to begin fishing for the day, and let it run until the camera’s batteries were exhausted. Battery runtime ranged from 33 to 92 minutes in length due to intermittent domestic electricity in the village and diminishing battery life over time. Fishers swam and searched for fish with their spearguns in front of them, producing a video in which most of the fish encountered and all of the fish pursued and captured were visible. Nine fishers recorded 18 videos totaling 18.5 hours of fishing footage. Each fisher took a camera between one and three times. We attempted to obtain videos from as many fishers as possible, while purposefully maintaining a balance of compressor divers and freedivers. Because participation in the study was voluntary, our sampling of fishers was not random. Some fishers declined to take the camera, and others took them but did not record useable videos. We obtained videos from fishers with a diversity of years of experience, age, and reputation as a fisher (Table A in S3 File). Therefore, the fishers in our sample encompassed a variety of fishing styles and skill levels found among fishers in this community, though we cannot claim they appropriately represent the larger population. Limitations of video sampling are addressed in the Supplemental Information.

We conducted content analysis [39] to extract data from the videos by coding the encounters a diver had with fish. We recorded characteristics of every fish seen (i.e. encountered) in the video that was a known harvested species and size (coded variables in Table 1). The videos did not show every fish that fishers saw, because fishers tend to search by turning their heads without always swinging the speargun and camera the same way. However, fishers typically fished by moving forward in one direction along a reef and scanning ahead. We cannot say what proportion of fish encountered actually appeared on video. Every fish that was pursued can be seen, but there were likely fish outside of the camera’s field of view that the fisher saw (i.e., encountered) and decided not to target. Nevertheless, the videos do provide direct observations of many of the fish fishers encountered and the decisions fishers made in the moment. Species identifications were made following a published field guide [40]. Each fish species was later categorized into broader taxonomic groups, largely based on fish families. One species, lionfish *Pterois volitans*, was considered separately, all invertebrates were lumped together, and ‘other’ was a catchall for rarely encountered families. When two or more fish were first observed in the video at approximately the same time (i.e. within a few seconds of each other), we considered them as encountered simultaneously in a single encounter event. Fishers saw 1374 harvestable fish or invertebrates within 804 separate encounter events and captured 289 individual fish or invertebrates.

**Table 1:** Fish attributes assigned to each fish observed in the fishing videos. Variable Type refers to the way each variable was included in statistical models of fishers’ selection decisions.

Variable	Variable Type	Description	Levels or Range	Relevant Information and Predictions of the Relationship with Targeting Decisions
Targeted	Response	Was a fish targeted by the fisher?	0/1 (no/yes)	Fishers’ decisions are most evident in what they target, not what they catch, because many other factors outside of the fisher’s intention affect what is finally caught.
Value	Predictor (continuous, fixed)	Estimated value of the fish in Dominican pesos, calculated based on the average weight of a fish of the assigned class multiplied by the price paid per pound	$DR 6–80 ($US 0.12–1.76)	Nearly all fish caught are sold, and their value in the market varies by species and size. Prediction: The greater the value of the fish, the higher probability it will be targeted.
Distance	Predictor (continuous, fixed)	Proximity of the fish to the fisherman when first encountered	1–3 (0-2m—>4m)	The proximity of a fish affects how much effort is required to catch it and how likely successful capture is. The farther away a fish is the more effort and greater likelihood the fish will escape. Prediction: Fishers should prefer closer fish.
Taxon	Predictor (categorical, fixed)	Factor levels approximately follow taxonomic groupings	parrotfish, snapper, grouper, grunt, lionfish, squirrelfish, invertebrates, other	There may be characteristics of each taxon that fishers prefer, but were not observed in this study. No clear *a priori* predictions.
Fisher	Predictor (random)	The identity of the spearfisher	9 fishers	Fishers’ decision making may vary by individual because of experience, ability, financial need, or any number of other personal attributes

We assigned every fish to a market class, which is a function of species and size (Table 2). We did not have a way to measure the exact size of fish. Instead, we used various indicators to estimate size as it corresponds to the different market classes. For example, initial phase redband parrotfish (*Sparisoma aurofrenatum*) are abundant in the environment and fishers’ catches and are only ever big enough to be third class fish (i.e. the least valuable class). Terminal phase redband parrotfish, on the other hand, might be third class if they are small or second class if they are large enough. Large terminal phase stoplight parrotfish (*S*. *viride*) are always big enough to be second class. Using these benchmarks, we were able to calibrate our visual size estimations from the videos much in the same way one does while conducting underwater visual censuses of reef fish [41, 42]. We included two in-between categories of market class, ‘first or second’ and ‘second or third’, for two reasons. First, it reflects the precision with which a fisherman can assess the market class of a fish while fishing. There is always uncertainty, even for an experienced fisherman, as to how a buyer will decide to classify a fish that is on the border between two classes. Second, it allowed us to confidently assign each fish to a category in a replicable way.

**Table 2:** The expected market value of a fish is calculated as the price per pound multiplied by median weight.

Market Class	Price units: pesos / lb	Weight per fish units: lbs median (1^st^–3^rd^ quartile)	Expected Value of one fish units: pesos price * med. weight
First	55	0.90 (0.75–1.17)	49.5
First or Second	47.5	0.69	32.775
Second	40	0.48 (0.42–0.56)	19.2
Second or Third	32.5	0.375	12.1875
Third	25	0.27 (0.25–0.31)	6.8
Lobster	70	0.69 (0.60–1.23)	48.3
Octopus	55	0.50 (0.34–0.87)	27.5
Conch	40	0.33 (*n = 1 batch weight)	13.2
Crab	25	3.00 (2.85–3.23)	75.0
Eel	15	1.13 (0.88–1.50)	17.0

The monetary value of a fish was determined by multiplying the median weight of cleaned fish (gutted and scales removed, head on) from each market class by the known price per pound of the respective market class in 2014, when this study took place. The monetary value of fish assigned to one of the in-between classes was estimated as the weight halfway between two classes multiplied by the price per pound halfway between those classes. In addition to finfish, there are several types of invertebrates harvested (i.e., lobster, octopus, conch, and crab), each with their own median weights and per-pound market prices. Further description of the market classification system can be found in the Supporting Information.

We estimated the distance at which a fisher first encounters a fish as being either 0-2m, 2-4m, or greater than 4m away. Similar to our estimations of fish size, we used objects of approximate known length to judge distances. Most fishers’ spears are approximately one meter long, providing a good yardstick from which to extrapolate. However, estimating distance from a two-dimensional video is imprecise. The inaccuracy of our measurements limits our ability to confidently assess the influence of distance upon encounter on fishers’ targeting decisions. Although our methods have captured this variable in coarse detail, our findings regarding distance should be considered approximate.

If multiple fish were encountered, we calculated several variables that, together, may help describe a fish’s desirability relative to other fish in the same encounter. The variables we calculated for fish within an encounter were as follows: the difference between a fish’s value and the most valuable fish; the difference in distance between a fish and the closest fish; and the difference in distance between a fish and the most valuable fish. We also noted the number of fish in the encounter. These variables are described in more detail in Table 3.

**Table 3:** Variables pertaining to encounters with multiple fish.

Variable	Variable Type	Description	Levels or Range	Relevant Information and Predictions of the Relationship with Targeting Decisions
Difference in value from most valuable	Predictor (continuous, fixed)	A fish’s value subtracted from the value of the most valuable fish in an encounter	$DR 0–68.25 ($US 0.00–1.50)	The value of a fish in relation to other fish may affect their likelihood of being targeted. Prediction: Fishers should prefer more valuable fish.
Difference in distance to fisher from closest	Predictor (continuous, fixed)	The proximity of the closest fish subtracted from the proximity of the fish in question	0–2 (same distance as closest fish—up to 4m from closest fish)	The distance between a fish and fisher in relation to other fish may affect their likelihood of being targeted. Prediction: Fishers should prefer closer fish.
Distance from most valuable	Predictor (continuous, fixed)	The proximity of the most valuable fish subtracted from the value of the fish in question	-2–1 (same distance as closest fish, up to 2m from closest, up to 4m from closest fish)	If the most valuable fish is farther away than other, less valuable fish, the fisher faces a tradeoff that this variable partly measures. Prediction: The closer a fish is to the fisher compared to the most valuable fish, the higher the probability the fish in question will be targeted.
Number of fish in encounter	Predictor (continuous, fixed)	The number of fish encountered at the same time	2–10 fish	Groups of fish may provide better or worse targets to fishers. Prediction: No *a priori* predictions

We also extracted data on fishers’ foraging behavior throughout each video. Behaviors were classified into four categories: searching, pursuing, capturing, and resetting. Pursuing was defined as beginning the moment a fisher targeted a fish, recognized by aligning himself with a fish and moving towards it, and ending when he either shot at the fish or stopped moving in its direction, in the event that no shot was taken. If a targeted fish was ultimately caught, capturing began immediately after the shot was fired and ended when the fish was secured on the fisher’s stringer and the fisherman returned to searching. If the fisher shot at the targeted fish but it ultimately escaped, we defined resetting as the time immediately after the failed shot was fired to the moment the fisher retrieved his spear and inserted it back into his speargun. Searching was defined as anytime the fisherman was in the water not engaged in any of the other behaviors. See the Supplemental Information for further explanation of the coding methods we used.

### Statistical methods

We analyzed fishers’ catch by describing the quantity and types of fish that fishers successfully captured while fishing. We calculated the number of fish fishers caught per hour of searching, and compared this rate between fishing methods, and across market classes and taxa. We tested for differences in mean catch rates between fishing methods and among taxa and market classes using two-way Analysis of Variance (ANOVA) for unequal variances. We used Tukey’s test *post hoc* to determine differences between levels of taxa and market classes.

We examined the opportunities fishers were presented with by examining the rate at which fishers encounter sellable fish. We again tested for differences between fishing methods and among taxa and market classes using two-way ANOVAs. We used Tukey’s test to determine differences between levels of taxa and market classes. We described and visually assessed the distribution of the number of fish in an encounter between fishing methods. We performed a non-parametric Wilcoxon test to compare the mean handling times of freedivers and compressor divers, because the data were strongly skewed. We used a t-test to compare how often pursued fish escaped capture between fishing methods.

We explored how fishers made targeting decisions by fitting decision models to our observations of the fish that were encountered and whether or not fishers targeted them. The encounter data were structured with each fish as a data point containing various characteristics of the fish, of the encounter, and of the fisher. We had three questions about the fishing process we wanted to address with the decision modeling: 1) Do fishers preferentially target parrotfish? 2)What is the effect of market value on targeting decisions? 3) Are there differences between the way compressor divers’ and freedivers’ make targeting decisions?

To answer these questions, we fit mixed-effects logistic regression models to these data. To analyze these data, first we separated the encounter data into encounters with single fish and encounters with multiple fish. We separated the data this way because they present fishers with two different decision scenarios. When only one fish is encountered, the fisher simply has to decide whether or not that fish is desirable enough to be worth the effort of pursuing it. When more than one fish is encountered, the fisher has to weigh the merits of each fish independently and in relation to other fish in the encounter.

We fit two models to the fish encounter data: one for single-fish encounters and another for multiple-fish encounters. We specified the single-fish model with whether a fish was targeted being predicted by a fish’s value, distance, and taxon, as well as the fishing method being used (Table 1). We specified the multiple-fish model with whether a fish was targeted being predicted by a fish’s value, value relative to the most valuable fish in the encounter, distance, distance relative to the closest fish in the encounter, and distance relative to the most valuable fish in an encounter, as well as the number of fish in the encounter and the fishing method being used (multiple-encounter variables in Table 2). We did not include taxon in the multiple-fish model because doing so caused convergence errors in the model fitting procedure. We also attempted to specify models that included interaction terms between variables. The value by taxon and distance by taxon interaction models for single-fish encounters failed to converge and were discarded. The value by distance interaction models for the single- and multiple-fish encounter models did converge, and the outcome is reported.

We then examined the parameter estimates and the confidence intervals for each variable to determine the direction and strength of each variable’s impact on targeting decisions. The encounter data were nested by the fisher being observed, so we included a random intercept called ‘Fisher ID’ in all models. This term accounts for fisher-specific preferences, tendencies, and abilities.

To determine whether there are differences between compressor divers’ and freedivers’ decision making, we fit another model for single- and multiple-fish encounters that included fishing method as a predictor variable. These models also included all of the previously-mentioned fish attributes. We then compared the models that included the fishing method variable to those without the method variable using a likelihood ratio test to determine whether there were systematic differences between the way compressor divers and freedivers make decisions.

The unit of observation for this analysis was each encountered fish (n = 1374). We excluded from this analysis simultaneous encounters with two or more fish of the exact same species, market value, and proximity. For example, if a fisher encountered a group of five, second class white grunts under the same coral head, we removed four of those fish from the decision making analysis. We did this because the fisher really only faced one decision for the purposes of this analysis: whether or not to pursue a second class white grunt at that distance. If the fisher later encountered another single fish or school of fish with the same characteristics, we considered this another encounter. Therefore, the datasets for this analysis only included 572 fish in single-fish encounters and 561 unique fish in multiple-fish encounters.

Coefficients of the variables are reported in units of odds. Coefficients with a value of one had no effect on the chances of a fish being targeted. Values greater than one increased the chances that a fish was targeted, and values less than one decreased a fish’s chances. For the continuous variables, a value of 1.10, would mean that for every one unit increase in the variable (e.g. one peso or one meter), the chances of a fish being targeted increased 10%. Variables whose confidence intervals do not overlap one had a reliably positive or negative effect on targeting decisions. We conducted all analyses in R [43] using the lme4 package [44] for mixed effects modeling and the ggplot2 package [45] for figures.

## Results

### Catch

**Fig 2:**
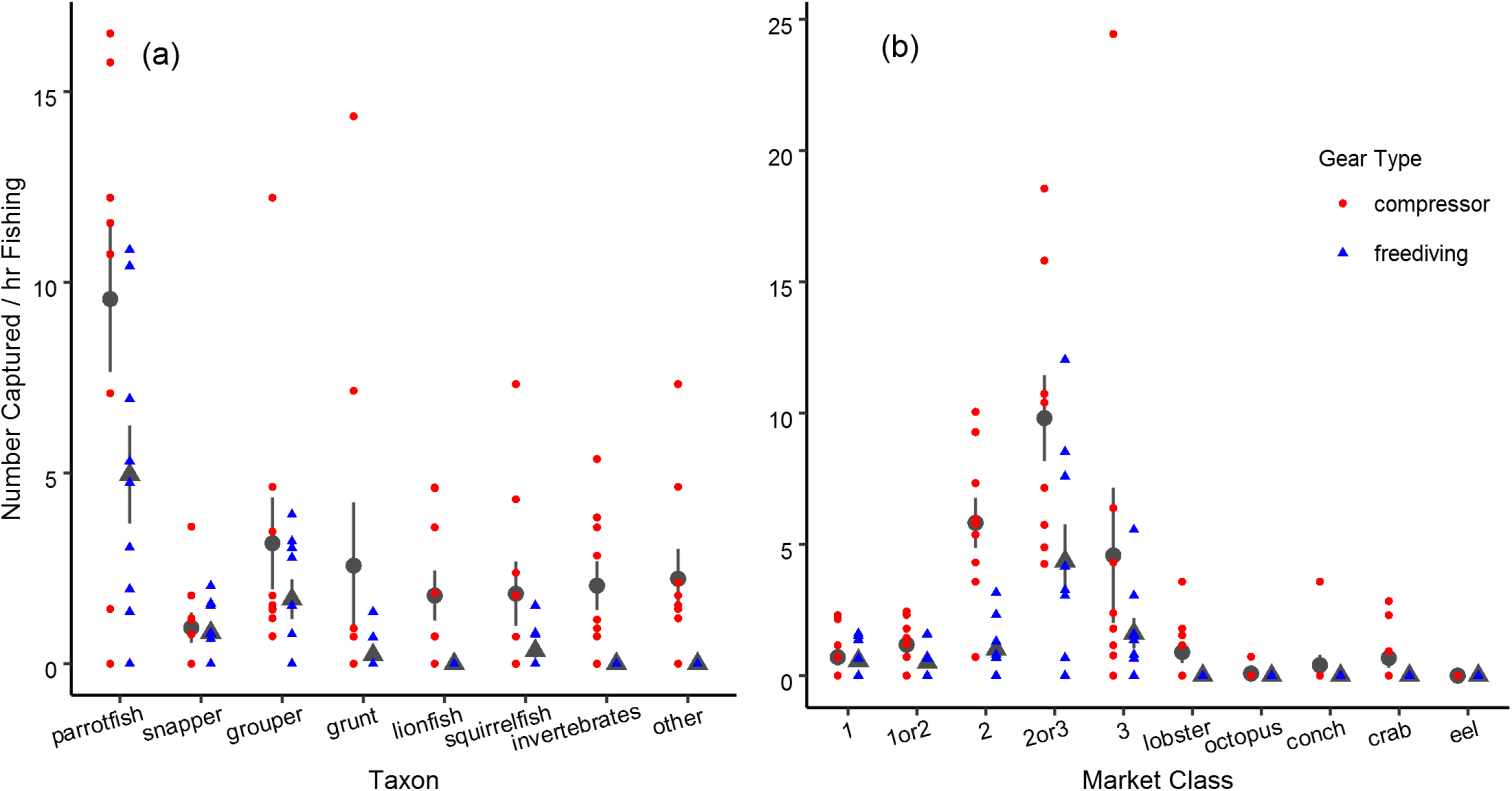
Fish captured. Capture rates varied by taxon (a) and market class (b), as well as between the two gear types. Points represent the capture rates from individual videos, dark gray symbols are the means, and error bars are 95% confidence intervals.

Fishers maintained the patterns in catch we had separately observed in this fishery (Fig 2 and S1 File; [46]), with important differences in capture rate between fishing methods (F(1,128) = 21.11, p < 0.001) and fish taxa (F(7,128) = 12.40, p < 0.001). Compressor divers generally caught more fish of all types than freedivers. The relative proportions of various taxa were similar for compressor divers and freedivers, as evidenced by the lack of a significant interaction between method and taxon (F(7,128) = 1.03, p = 0.414). However, there was more variability in the number caught by compressor divers (Fig 2A). Parrotfish were the most frequently caught taxon for both gear types. Parrotfish capture rate was significantly different than all other taxa, and none of the other taxa were statistically different from one another. Compressor divers and freedivers caught different numbers of fish of each market class (method by taxon interaction: F(9, 160) = 3.11, p = 0.002). Compressor divers caught more second and second-or-third class fish, whereas freedivers caught mostly second-or-third (Fig 2B).

### Opportunities spearfishers experience

**Fig 3:**
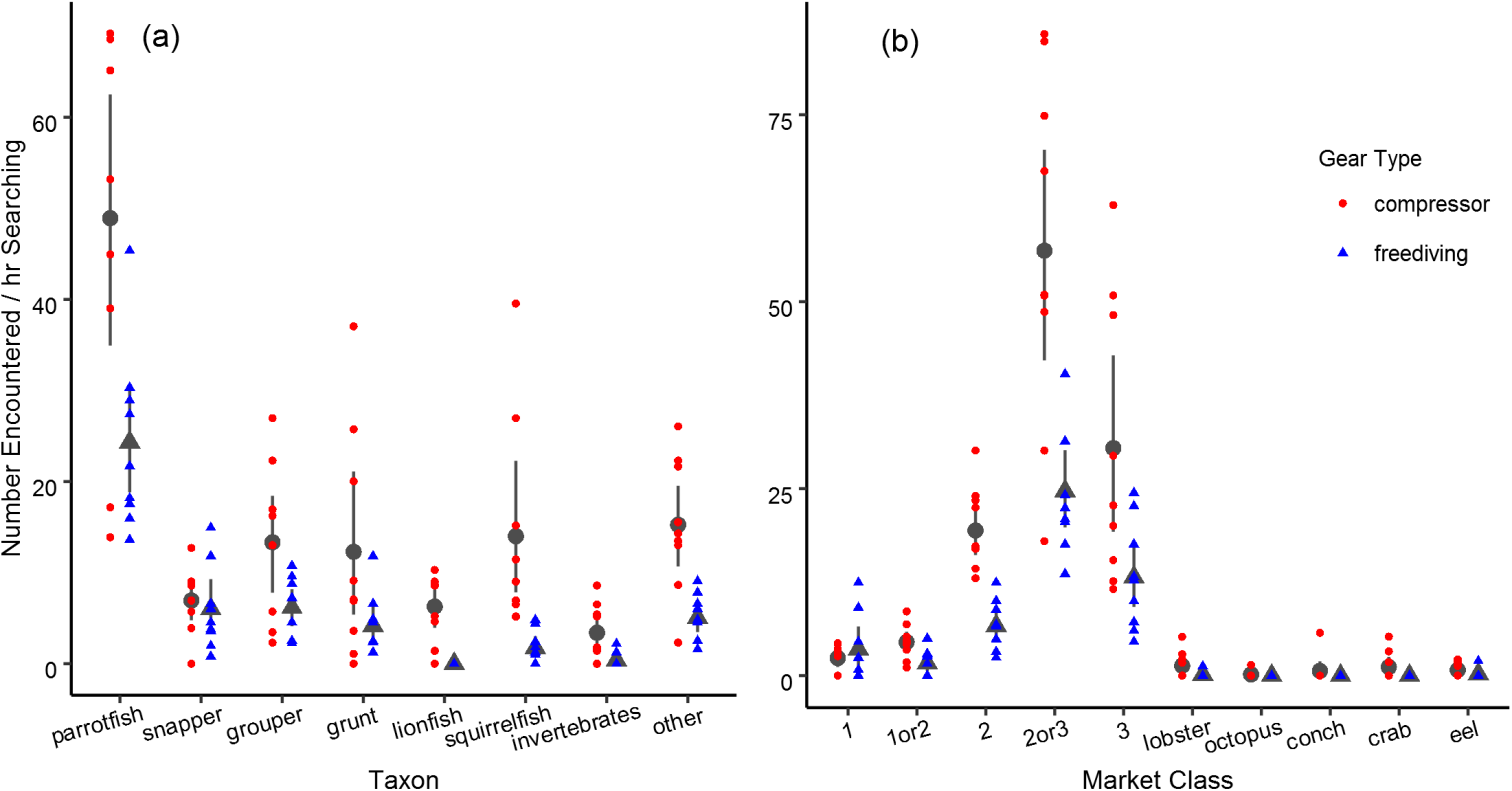
Fish encountered. Encounters with fish varied by taxon (a) and market class (b) as well as between the two gear types. Points represent the encounter rates from individual videos, dark gray symbols are the means, and error bars are 95% confidence intervals.

The encounter rate of all sellable fish varied substantially between compressor and freediving (t(16) = 17.17, p < 0.001). Compressor divers on average saw 120 fish per hour of searching (sd = 28.8), whereas freedivers only encountered 48 fish per hour of searching (sd = 9.6). Encounter rates are broken down by taxon and market class in Fig 3. Encounter rates with the various taxa differed between fishing methods (method by taxon interaction: F(7, 128) = 3.47, p = 0.002). By taxa, fishers encountered parrotfish more frequently than any other fish (Fig 3A). Fishers using either method also most frequently encountered fish between second and third class (Fig 3B).

### Constraints associated with spearfishing

**Fig 4:**
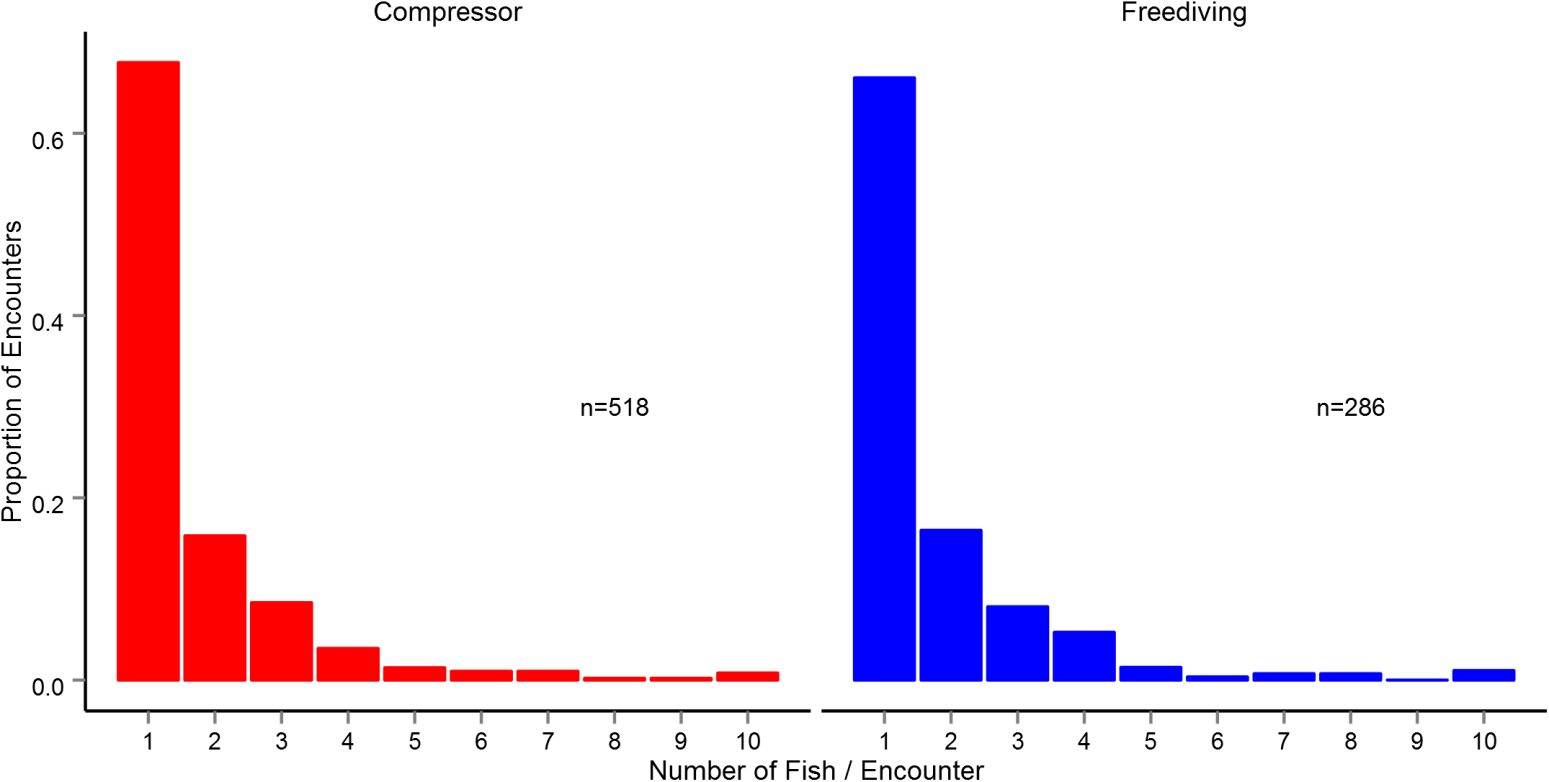
Number of fish encountered at the same time. Compressor divers and freedivers encountered mostly single fish. One third of encounters were with two or more fish.

Fishers cannot catch every fish they come across; we identified three constraints that narrow fishers’ opportunities. First, fishers sometimes encounter multiple fish simultaneously, and if one is pursued, the rest typically flee and become unavailable to the fisherman. Therefore, the number of fish that a fisherman can catch is set by the number of encounter events, be it with one or more fish, not the total number of fish seen. For both compressor divers and freedivers, approximately one third of encounters were with more than one fish. Sixteen percent of encounters were with two fish, and encounters with three or more fish made up the remainder (Fig 4). This first constraint reduced the mean rate of encounter events to 52 per hour of searching (sd = 20.6) for compressor divers and 19 encounters per hour of searching (sd = 6.3) for freedivers, a significant difference between methods (t(16) = 4.01, p = 0.002).

Another constraint evident in the fishing videos is that some targeted fish get away. Fish escaped while fishers were pursuing them, some shots missed their target, some shots hit the target fish but did not penetrate their bodies, and some fish even escaped after being completely impaled by the spear. Most fishers did not use barbed spears, which allowed otherwise trapped fish to wriggle free. Compressor divers caught more of the fish they targeted than did freedivers (t(16) = 3.47, p = 0.004). Of all fish targeted, compressor divers caught 66% (sd = 0.14) whereas freedivers caught 39% (sd = 0.19).

**Fig 5:**
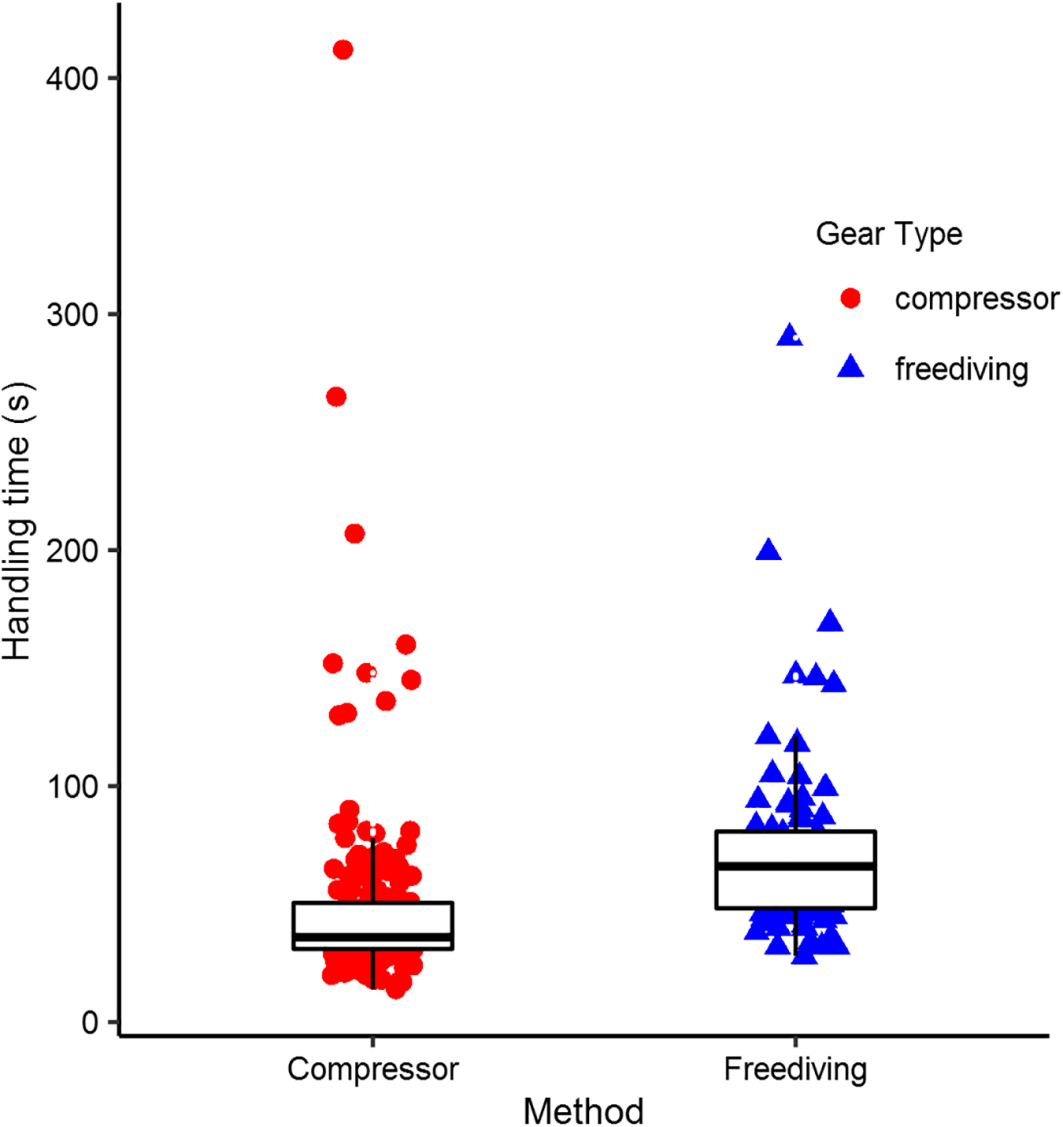
Handling time. The time required to pursue and capture a fish, i.e. ‘handling time,’ varied by fishing method. Points are individual fish captures; boxplots show the median, first and third quartiles, and 1.5 times the inter-quartile range.

The final constraint we identified is the amount of time it takes to pursue, capture, and reset for another pursuit. These activities constitute prey handling time [47], and they represent time that cannot be dedicated to searching for other prey. The duration of handling time thereby reduces the number of potential prey items a fisherman encounters. Handling times ranged from 14 to 412 seconds. The distribution was heavily right skewed, with many short handling times and a few very long handling times, for both compressor and freedivers (Fig 5). A non-parametric Wilcoxon test shows that handling times differ by fishing method (W = 3443.5, n_compressor_ = 198, n_freediving_ = 90). For compressor divers, the median handling time was 36s (1^st^ and 3^rd^ quartiles: 31, 48). Freedivers required nearly twice as much time to handle their prey, with a median of 66s (1^st^ and 3^rd^ quartiles: 51, 81).

We estimated how many fish a fisherman could maximally catch in an hour by resampling handling times from each method’s distribution until they summed to one hour, and repeated this procedure 1000 times. If fishers could continuously catch fish without having to spend time searching for them, we estimate compressor divers could pursue on average 75 fish in an hour (sd = 7.0) and freedivers could pursue 50 fish in an hour (sd = 3.8). Combined with their respective percentages of successful captures reported above, we estimate compressor divers could catch approximately 50 fish per hour and freedivers 20 fish per hour if they did not have to spend time searching.

### Fisher decision making

**Fig 6:**
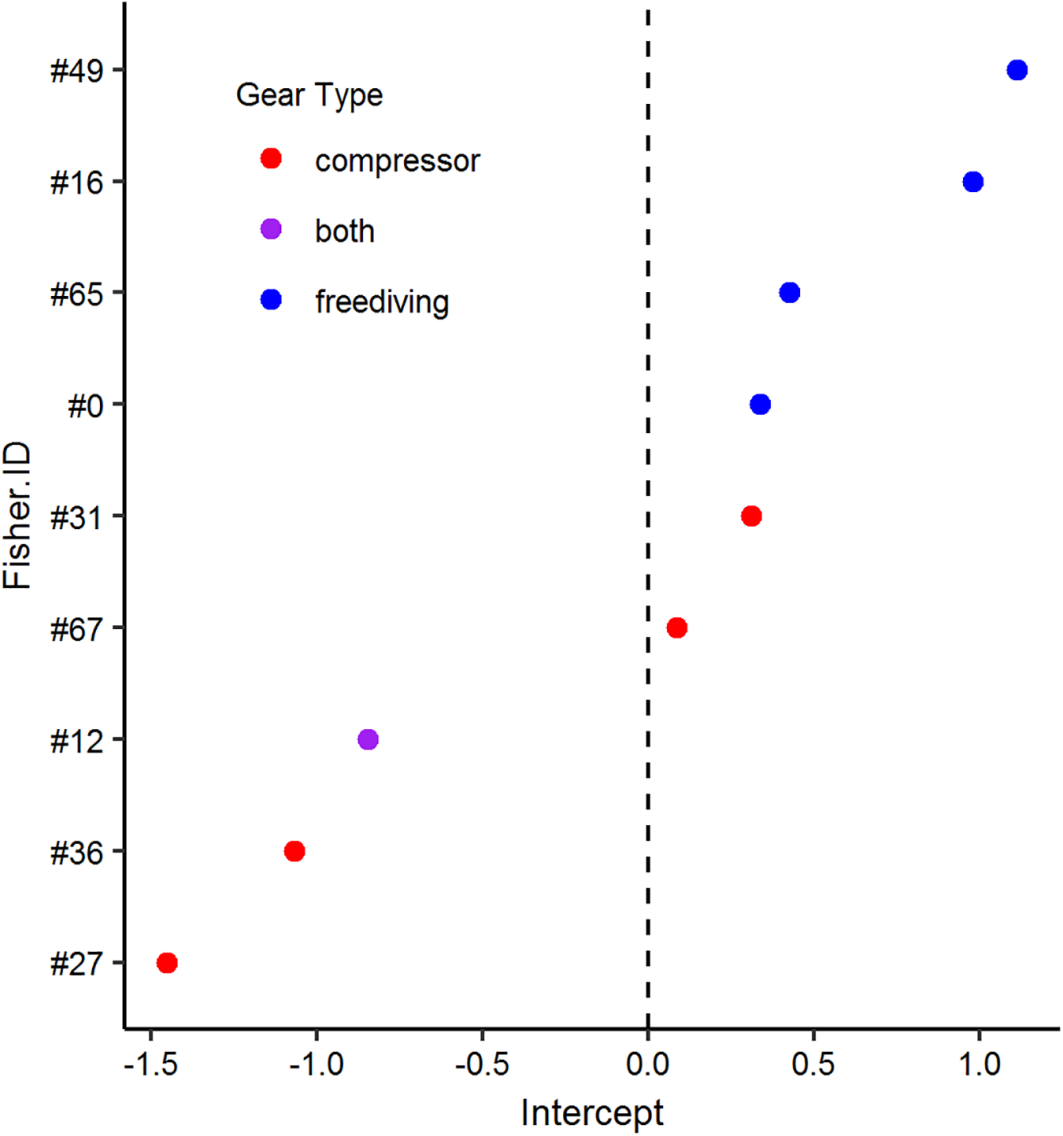
Random intercept values. The estimates of the random intercept variable, fisher identity, reflect a fishers’ overall tendency to pursue the fish they encounter.

Compressor divers and freedivers made targeting decisions in much the same way. Including fishing method did not improve fit in either of the decision models (single-fish encounters: F(df = 1) = 0.264, p = 0.607; multiple-fish encounters: F(df = 1) = 0.536, p = 0.464). However, exploring the random effect of fisher identity suggested that freedivers may be more likely overall to target fish than compressor divers. Freedivers tended to have higher estimated intercepts within the random effect, meaning they had a generally higher likelihood of going after a fish than compressor divers (Fig 6). Perhaps fishing method would have provided a statistically significant effect had we left out the random effect of fisher identity, though we cannot say for sure. However, we choose to retain the random effect variable because of the nested structure of the data. Here, we present the decision models without fishing method as a predictor variable.

Fishers considered a fish’s value, distance upon encounter, and taxon when deciding whether or not to target and pursue fish when only one fish was encountered (Table 4). Value and distance had highly significant effects in the expected direction. Fishers were more likely to target fish when they were more valuable and closer.

**Table 4:** Parameter estimates (in odds), confidence intervals, and p values for the fixed effects (fish traits only) in the fisher targeting decision model when only one fish was encountered. Coefficients were transformed from log-odds to odds by exponentiating the original estimates.

Variable	Coefficient (95% CI)	Prob. > |Z|
(intercept)	5.15 (1.97–13.45)	< 0.001 ***
Value	1.11 (1.07–1.15)	< 0.001 ***
Distance	0.38 (0.28–0.51)	< 0.001 ***
Taxon–Lutjanidae (snapper)	0.20 (0.08–0.54)	0.001 **
Taxon–Epinephelinae (grouper)	0.39 (0.21–0.73)	0.004 **
Taxon—Haemulidae (grunt)	0.60 (0.29–1.24)	0.167
Taxon–*Pterois volitans* (lionfish)	0.33 (0.13–0.83)	0.018 *
Taxon–Holocentridae, Priacanthidae (squirrelfish, bigeyes)	0.46 (0.19–1.08)	0.075
Taxon—invertebrates	0.39 (0.05–3.46)	0.401
Taxon–other	0.21 (0.10–0.44)	< 0.001 ***

Footnotes:

*** p < 0.001

** p < 0.01

* p < 0.05

When multiple fish were encountered, fishers faced a more complex decision, but market value and distance still played important roles in whether or not a fisher pursued a fish. Fishers were more likely to target higher value fish and those that were closer than others (Table 5). Absolute distance was unimportant in this model, but the distance between a fish and the closest fish did significantly affect decisions. The distance between a fish and the most valuable in the encounter seemed to have little effect. The number of fish in an encounter reduced the probability of being targeted for any individual fish. Fishers might prefer to attack groups of fish over single fish, something we did not evaluate here. If this is the case, the fact that fishers can only target one fish from a group automatically reduces each individual’s chances of being pursued. The only interaction that did not cause convergance errors was the value by distance term; it was not significant in either case (single-fish model: p = 0.236, multiple-fish model: p = 0.324).

**Table 5:** Parameter estimates (in odds), confidence intervals, and p values for the fixed effects in the fisher targeting decision model when multiple fish were encountered at the same time. Coefficients were transformed from log-odds to odds by exponentiating the original estimates. Taxon coefficients correspond to dummy variables for different factor levels, and are relative to the reference level: Taxon–Parrotfish.

Variable	Coefficient (95% CI)	Prob. > |Z|
(intercept)	0.55 (0.24–1.27)	0.164
Value	1.08 (1.05–1.12)	< 0.001 ***
Difference in value from most valuable	0.95 (0.91–0.98)	0.005 **
Distance	0.81 (0.59–1.12)	0.209
Difference in distance to fisher from closest	0.48 (0.27–0.85)	0.011 *
Distance from most valuable	0.79 (0.51–1.24)	0.310
Number of fish in encounter	0.77 (0.61–0.97)	0.027 *

Footnotes:

*** p < 0.001

** p < 0.01

* p < 0.05

The estimated coefficients describe the direction and strength of each variables’ influence on targeting decisions. The coefficient for value, 1.11 for single-fish encounters and 1.08 for multiple-fish encounters, implies that a fish’s chances of being targeted increase between 8 and 11% for every additional peso they will fetch when sold. The average difference in value between second and third class fish is 15 pesos (Table 2), meaning second class fish are 180% as likely to be targeted than third class fish, all else being equal. This effect is affected by the values of other fish encountered at the same time, if any. The coefficient for relative value, 0.95, implies that a fish’s chances of being targeted decrease 5% for every peso of lower value than the most valuable fish in the encounter. Regarding distance, for every two meters away from a fisher that a fish was encountered, the model of single-fish encounters estimates the chances of being targeted drop by 62%. If there is another closer fish in the same encounter, the chances of being targeted drop by 52% for every meter farther away a fish was. This metric of relative distance appears influence fishers’ decisions more than absolute distance, as the ‘Distance’ variable alone was not significant in the decision model. The distance between a fish and the most valuable fish in an encounter was not statistically significant in the model.

We compared the effect of all other taxa to parrotfish on fishers’ targeting decisions because parrotfish are the most frequently caught taxon and are particularly ecologically valuable [48]. Fishers targeted parrotfish more than snapper, grouper, lionfish, and ‘other’ taxa (Table 4). Therefore, parrotfish were more likely to be targeted than these taxa after accounting for differences in price and proximity. There was no statistical difference between targeting of parrotfish versus grunts, squirrelfish/bigeyes, or invertebrates at the p < 0.05 level. Note that the effect of squirrelfish/bigeyes was nearly significant with a p-value < 0.10.

The variables in the encounter data were generally not highly correlated. In the single-fish encounter data, the strongest correlation was between ‘Taxon-snapper’ and ‘Value’ at rho = -0.505; the snappers fishers encountered tended to be small and not very valuable. This leads us to believe that snappers might not be selected against quite as strongly as the model suggests, i.e., 82% less likely to be targeted than an equivalent parrotfish. The rest of the correlations between fixed effects were between 0.246 (‘Taxon-other’ and ‘Value’) and -0.011. For the multiple-fish encounter data, the largest correlation was between ‘Distance’ and ‘Difference in distance to fisher from closest’ at rho = -0.308. The correlation between ‘Value’ and ‘Difference in value’ was 0.214.

## Discussion

The video observations we obtained gave us a glimpse into the underwater world fishers experience. This allowed us to quantify fishers’ opportunities to catch fish, the factors limiting their catch, and the way they chose which fish to target. Data collection from video observations required more time and effort to conduct and analyze compared with survey interview techniques, which could be another means of gathering information on the fishing process. However, only observing fishers as they fish, from their perspective, allowed us to quantify the variables we examined, such as the encounter rate of fisher with various types of fish, how often fishers fail to catch the fish they pursue, or the magnitude of the effect of market value on fishers’ targeting decisions. Furthermore, we could not have reliably contrasted these variables between fishing methods using interview methods. The results have implications for two management interventions that are under active consideration in Buen Hombre, as well as the broader Caribbean. These are gear restriction, specifically banning compressor diving, and controlling the species composition of the catch, specifically prohibiting capture of parrotfish [49,50]. Here, we discuss what we learned about fishing with these two gear types, how parrotfish compare to other species in the fishery, and implications of these insights for fisheries management strategies in this and other Caribbean reef fisheries.

### Compressor diving, freediving, and the implications of gear restriction

Compressor divers in this study encountered more fish than freedivers. The higher encounter rate was likely due to compressor divers’ ability to effectively fish on deeper reefs than freedivers. The introduction of compressor diving in the early 1990s gave fishers the ability to access much deeper habitats than had previously been possible [51]. Freedivers typically stay in areas 2-10m deep, whereas compressor divers typically fish areas 10-30m deep. These deeper reefs currently hold more fish either because they are naturally more productive, or because they have received and continue to receive less fishing pressure. Regardless of why the difference in fish encounters exists, the increased opportunities for compressor divers set the stage for these fishers to catch more fish than their freediving colleagues.

While the number of encounters differed substantially between compressor and freedivers, the relative proportion of encounters with each taxa, market class, and group size was quite similar (Figs 3 and 4). Still, compressor divers did see a greater number of encounters with higher quality fish because they saw more of all types of fish.

Compressor diving is also a more effective method for capturing fish. Compressor divers captured 69% of the fish they targeted, 28% more than freedivers. Compressor divers also spent less time catching each fish, which allows a greater capture rate. If unlimited prey was available and fishers did not have to spend time searching for the next fish, compressor divers would be able to harvest 50% more fish than freedivers. This is largely due to compressor divers being able to fish continuously without the need to surface for air. In contrast, freedivers must descend from the surface to approach each fish, pursue and capture it, and then return to the surface to breathe. Further, the time freedivers spend returning to the surface is lost time that compressor divers get to spend searching for the next fish. Even if both types of divers encountered the same number of fish, we would expect compressor divers to catch more of them.

Despite the differences in encounter rates and the efficacy of each method, we found no significant difference in the way fishers made targeting decisions. We originally hypothesized that compressor divers’ decisions would be less affected by distance than freedivers’ because they can travel long distances underwater without needing to surface. We also expected compressor divers to show stronger preferences for more valuable taxa and market classes. However, adding fishing method to the targeting decision models did not help explain fishers’ behavior. One explanation could be that there is substantial variation in the behavior of individual fishers within each fishing method. Anecdotally, we know this is true of the fishers in Buen Hombre; some freedivers catch a greater proportion of higher class fish than some compressor divers. Further, Fig 6 shows individual-level variation in fishers’ propensity to pursue the fish they encounter, which is one aspect of fishing behavior. Nevertheless, all fishers in this community operate within the same market context, and fishers encounter very similar proportions of each taxa and market class. Therefore, the differences in catch characteristics between compressor divers and freedivers are the result of differences in the number of opportunities and the types of constraints the fishers have, not a substantially different decision making process.

Dominican fishing law 307–04 prohibits the use of compressors while fishing [52], though this law is not currently enforced in Buen Hombre. Effectively banning compressor diving would reduce the lethality of spearfishing and, therefore, decrease total catch, at least in the short term. Even if all compressor divers began freediving, the decreased ability to access deep reefs and the increased constraints on the number of fish that can be caught would reduce overall harvest unless fish populations rebuild and become more productive. A potential downside to banning compressor diving is that fishing pressure on the already degraded shallow reefs could increase, potentially causing further harm to these areas. The establishment of deep water refuges for reef fish, which may act as *de facto* no-take zones, would counteract the increased fishing pressure on shallow reefs to some extent. These zones would likely enhance larval export and spillover of adult fish to shallow reefs [53,54]. The net effect on fish densities in shallow areas should be studied further if compressors are eliminated. Nevertheless, prohibiting compressors should increase overall reef fish densities and biomass in this area.

In addition to meeting ecological goals, managers need to consider the social implications of a ban on compressor diving. Whether or not compressor divers would transition to freediving would determine part of the social impact of eliminating compressor diving. Most compressor divers started fishing as freedivers, but many claim they would not be able to revert back. Additionally, for every one or two compressor divers there is a ‘*yolero*’, a person who tends the boat and compressor while fishing, whose livelihood also depends on this fishing method. Banning this technology would equally or more negatively affect yoleros compared to compressor divers themselves. Yoleros are often older fishers who no longer dive or are younger men who did not become divers if diving did not suit them. Most yoleros could not become divers, and the fishery would sustain the livelihoods of fewer people. An important difference between yoleros and divers, however, is that divers assume all of the risk associated with diving with compressed air. People in Buen Hombre and other tropical small scale fisheries die, become paralyzed, and disabled due to decompression sickness and embolisms from unsafe diving practices [36,55,56]. Additionally, more mild symptoms are a regular occurrence. Most people who currently oppose compressor diving in Buen Hombre do so because of the risk to human life. Stakeholders looking to garner support for eliminating this fishing method should highlight the benefits for human health and long-term fishery productivity to counter the short-term disruption for those who currently use compressors.

### The role of species identity in the spearfishing process

Spearfishers in Buen Hombre caught many different species of fish and invertebrates, though the frequency of capture varied substantially between taxa. Fishers’ catch appeared to reflect what they encountered, as assessed by visually comparing encounter rates to capture rates for the various taxa and market classes (Figs 2 and 3). Fishers, however, preferred certain taxa over others when making targeting decisions. The influence of three taxonomic groupings could not be distinguished from the effect of parrotfish, while four groupings had a negative effect. Grunts were the most similar in terms of market class and desirability. Like parrotfish, they can be second or third class, but never first. They also achieve roughly similar sizes as parrotfish. Invertebrates most likely could not be distinguished from parrotfish because this grouping included everything from highly valuable lobster to moderately valuable spider crab, and there were relatively fewer encounters with these species. Bigeyes and squirrelfish, the final non-significantly different taxonomic group, were always the third, least valuable market class. Further, the estimated effect of this taxon was quite negative (0.46), and nearly statistically significant (p = 0.075).

Surprisingly, fishers targeted snapper and grouper less than parrotfish. We had expected these species to receive more pressure because they can be the most valuable fish in the market, if they are big enough. We predicted fishers to always pursue those species that have a chance at being first class. Although the results of this study cannot explain why snappers and groupers were targeted less than parrotfish, interviews with fishers might be able to provide additional information on this behaviour. For example, the selection against snapper and grouper could come from fishers passing on smaller individuals of these species in favor of others of the same size that could never become first class fish, such as parrotfish, grunts, and squirrelfish/bigeyes.

The effect of taxon could also be driven by differences in escape behavior of the fish, the way fish swim, the group size in which fish are commonly encountered, or some other attribute of fish [57]. All of these characteristics have implications for the likelihood of capture, something that fishers would be aware of and likely consider. If these factors influence fishers’ targeting decisions, fishers essentially calculate an expected value of each fish; how much it is worth adjusted by the probability it will get away. Alternatively, taxon effects on targeting decisions could come from attributes affecting a fish’s non-monetary desirability to the fisher. Examples might be the ease of cleaning fish for sale, a fisher’s personal ability or habit of pursuing a given taxon, or some other preference or experience that fishers have but that this study and prior work [36] did not reveal.

If the influence of taxon seen in the decision model comes from consumer preferences, at least in part, consumer demand could be an effective means of changing fishers’ catch composition. Stakeholders hoping to alter the composition of the catch could attempt to create beliefs in the minds of fishers or consumers for certain types of fish and stigmas against others. For example, if fishers and consumers believed that parrotfish play an important role in maintaining good ecosystem condition, this could establish a social stigma and negative incentive to catching them. A public awareness campaign is already underway by Reef Check–Dominican Republic using social media and a restaurant certification scheme aimed at pressuring fishers and fish buyers to improve the ecological soundness of fishing, including reducing or eliminating parrotfish harvest (R. Torres, Reef Check–Dominican Republic, personal communication). Further discussions with fishers regarding preferences for certain taxa would complement this study.

The conditions already exist for price incentives to work; price influences fishers’ selection decisions, and fishers sometimes have to choose between parrotfish and other taxa. This suggests that price incentives for non-parrotfish likely would decrease the amount of parrotfish harvested. All else held equal, reducing the price of an average sized parrotfish by one peso (US$0.022 at the time of writing), equivalent to reducing the price per pound by two to four pesos, would reduce each fish’s chances of being pursued by around 11% (Table 3). Further, altering the prices of one type of fish also changes the relative value of each fish, when more than one is encountered at the same time, and would enhance selection pressure against parrotfish. Eco-labeling or controlled access to higher-value markets are possible mechanisms for creating a price incentive structure (Frederick Payton, executive director of AgroFrontera, a Dominican NGO working in the area, personal communication).

Studying individual fishers’ foraging behavior while underwater provided insight into how species restrictions would affect spearfishers’ opportunities and, therefore, catch. For example, prohibiting parrotfish capture would be the most direct way of reducing their catch and protecting their populations. Encounters in which no taxon other than parrotfish was present accounted for 40% of compressor divers’ and 50% of freedivers’ opportunities. Therefore, a ban on parrotfish would take away up to half of the opportunities spearfishers currently have to catch fish. The effectiveness of a parrotfish ban would depend on many aspects of monitoring, enforcement, and community engagement that are beyond the scope of this study. We cannot say how increased pressure on other species would affect those species’ populations and overall reef health. Other taxa could play other critical roles in reef ecosystem functioning that we do not yet fully understand, and we should take care not to create new problems through unintended consequences of management decisions [58].

### Future research

The effect of escape behavior and distance on fish-fisher dynamics should be explored further. Here, we showed that the distance between fish prey and spearfishers influences fishers’ decision making, but we do not know how it affects the probability of capturing fish. Other researchers have demonstrated differences in fish reactions to divers along a gradient of spearfishing pressure [57,59], but also did not quantify the impact on capture rate. Understanding the relationship between fish behavior and catchability would give us an idea of how behavioral adaptations do or do not protect them from fishing.

One factor that may have important consequences for fishery dynamics and management is individual-level variation between fishers [5]. Our analysis of the random variable fisher identity, and our experience observing and speaking with fishers and collecting data on the fishery, suggests that all fishers are unique in their abilities and foraging strategy. For instance, one freediver in the community consistently catches larger, more valuable fish than some compressor divers in contrast to the overall trend (personal observation). New interventions or policies will undoubtedly impact some individuals more than others, which will have consequences for the effectiveness and perception among fishers of management. Although it is not realistic to understand individual-level impacts in most cases, researchers and managers should be mindful to consider heterogeneity among fishers and gear types.

Deconstructing the process of fishing from the fishers’ perspective can help fisheries researchers understand social-ecological interactions and identify leverage points for management. Researchers should describe the context of opportunities and constraints fishers face because this bounds how fishers can react to changes in the governance system or the state of the resource. This creates the required starting point for understanding how fishers make decisions. Important decision making occurs at multiple temporal scales and at multiple points within the fishing process, and scientists should match their observations and analysis appropriately. Fishery analysts might be able to create more effective management interventions and better predict their outcomes if they invest the necessary effort into understanding the fishing process as experienced by fishers [60].

In this study, we observed fishers behavior at the smallest time scale on which they make decisions.

By the time fishers are in the water fishing, they have already made several decisions that have the ability to affect catch. At the most basic level, the person decided to fish for a living. People in fishing communities often have few livelihood options, though fishing is rarely the only option. Another level up, the fisher decided to go fishing on the day in question. Fishers in Buen Hombre are not obligated to go to sea every day. Had the fisher not been out that day, they would not have caught any fish. Finally, the fisher decided where he was going to fish on that day. Fishers here describe a very loose system in which they try not to revisit the same area frequently, but there is no fixed system of assigning fishers to fish a certain place. All of the reefs in the area may be of similar quality; we would expect a more established means of controlling access if some parts of the fishery area were much better than others. Still, site selection at least influences the specific opportunities a fisher has on any given day. All of these decisions, from choosing a profession to site selection, should be investigated further to better describe the determinants of fishing pressure in the system and, by extension, know how to intervene to reduce fishing pressure.

## Supporting information

S1 File Data, encounters with fish. These are the data derived by coding videos of fishers fishing. Each row is a fish that was observed in the videos, and columns report characteristics of the fish and the encounter.
(XLSX)

S2 File Data, video information. This file contains descriptive information of the videos of fishers fishing. Each row is a video, and rows contain characteristics of when the video was recorded and the fisher who did the recording.
(XLSX)

S3 File Supporting information. This document contains supplemental material referenced in the manuscript.
(DOCX)
