# Supplementary material for: Understanding spearfishing in a coral reef fishery: Fishers’ opportunities, constraints, and decision-making
Source: PLoS One. 2017 Jul 27;12(7):e0181617. doi: 10.1371/journal.pone.0181617 (PMC5531497; doi:10.1371/journal.pone.0181617)
Supplement: S3 File — This document contains supplemental material referenced in the manuscript. (DOCX) [file pone.0181617.s003.docx]

**Understanding spearfishing in a coral reef fishery: fishers’ opportunities, constraints, and decision-making**

**Supplementary Information**

***Fisher Information***

Fishers in this study were of diverse ages and years of fishing experience. They have also participated in landing surveys (unpublished), and their average daily catch is reported in table S1. Compressor divers typically caught more fish, but there is substantial variation within gear types.

**Table A:** Attributes of the fishers who participated in this study, and information independently collected on their daily catch.

| **ID #** | **Method** | **Videos** | **Total minutes** | **Age** | **Years fishing** | **Mean daily catch weight**  **(lbs cleaned fish)** | **Daily catches observed** |
| --- | --- | --- | --- | --- | --- | --- | --- |
| 27 | Compressor | 2 | 136 | 27 | 14 | 75.22 | 4 |
| 67 | Compressor | 2 | 110 | 42 | 20 | 75.50 | 8 |
| 31 | Compressor | 2 | 105 | 20 | 9 | 49.81 | 6 |
| 36 | Compressor | 2 | 94 | 31 | 15 | 40.78 | 5 |
| 12 | Compressor  Freediving | 1  2 | 84  128 | 29 | 12 | 45.32  25.23 | 2  10 |
| 16 | Freediving | 2 | 165 | 29 | 12 | 23.57 | 14 |
| 65 | Freediving | 3 | 244 | 19 | 7 | 18.93 | 10 |
| 49 | Freediving | 1 | 41 | 34 | 1 | 17.50 | 2 |
| 0 | Freediving | 1 | 33 | 16 | 3 | 13.88 | 4 |

***Market classes***

Fish are bought and sold according to their assigned market class, which is a function of species and size. Some species are only ever third class (the least valuable), others can be second or third class, and others can be first (the most valuable), second, or third class. For those species that can be multiple classes, the size of the individual fish determines which class it is assigned to; larger fish are classified into more valuable classes than smaller fish. There is no explicit rule dictating size bins for each market class, and fishers and buyers frequently dispute how fish are sorted, but sizes are quite consistent.

**Table B:** Fish taxa can be classified as one, two, or three market classes. In the case of multiple possible classes, larger individuals are placed into more valuable classes (1>2>3).

| **Family** | **Market Class or Classes** |
| --- | --- |
| Lutjanidae (snapper) | 1, 2, 3 |
| Epinephelidae (grouper) | 1, 2, 3 |
| Haemulidae (grunt) | 2, 3 |
| Scaridae (parrotfish) | 2, 3 |
| Scorpaenidae (lionfish) | 2, 3 |
| Sphyraenidae (barracuda) | 2, 3 |
| Scombridae (jacks and mackerels) | 2, 3 |
| Holocentridae (squirrelfish) | 3 |
| Priacanthidae (bigeye) | 3 |

We used videos of the process of sorting and weighing fishermen’s catches to estimate the average weight of fish in each class. We recorded and analyzed 71 videos for the number and species of fish in each market class. We found the median weight of individual fish by dividing the batch weight of each market class by the number of individual fish included in the weight. We then multiplied the median weight by the known price per pound to determine a typical value for a single fish of each market class. This information is presented in the manuscript in Table 2.

***Bias***

One way of checking whether fishermen fished differently with the camera than they normally do is to compare what was harvested in the videos and our data on what fishermen have caught otherwise. Below is a figure of fishermen’s daily harvests, broken down by market class (top) and taxon (bottom) during the summers of 2013 and 2014. This can be compared to the catch data from the fishing videos, shown in figure 1. The patterns in relative composition are qualitatively similar to the patterns of harvest we observed in our videos, suggesting that using the camera did not drastically change the way fishermen fished.


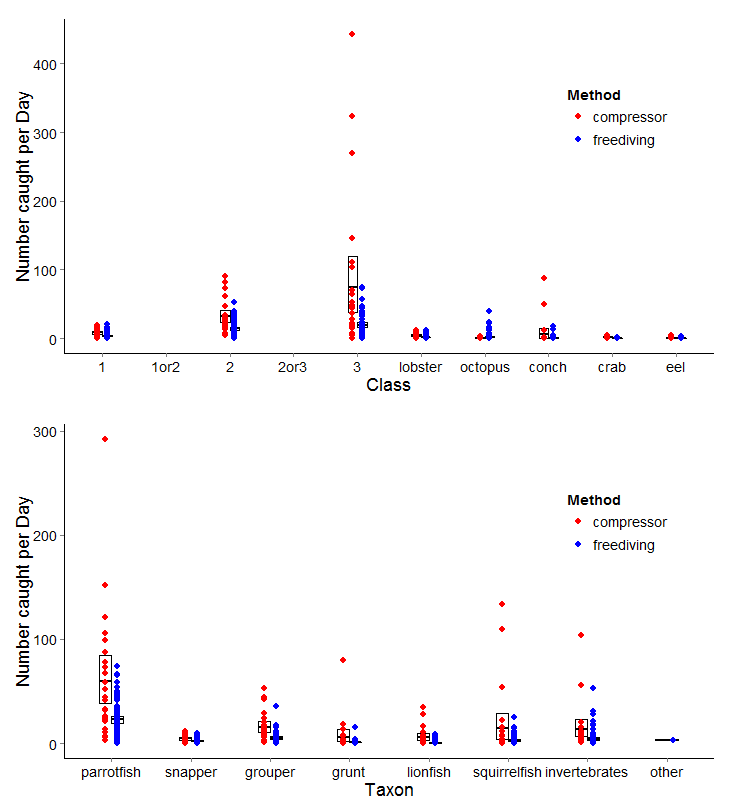


**Figure A:** Fishers’ daily catch by market class (top) and taxon (bottom), based on creel surveys at the landing point in Buen Hombre, showed similar patterns to the catch observed in the videos in this study. Center lines are means and boxes are standard deviations.
